# High Pressure (HP) in Spark Plasma Sintering (SPS) Processes: Application to the Polycrystalline Diamond

**DOI:** 10.3390/ma15144804

**Published:** 2022-07-09

**Authors:** Jérémy Guignard, Mythili Prakasam, Alain Largeteau

**Affiliations:** CNRS, Bordeaux INP, ICMCB, UMR 5026, University of Bordeaux, F-33600 Pessac, France

**Keywords:** high pressure, Spark Plasma Sintering, binderless diamonds

## Abstract

High-Pressure (HP) technology allows new possibilities of processing by Spark Plasma Synthesis (SPS). This process is mainly involved in the sintering process and for bonding, growing and reaction. High-Pressure tools combined with SPS is applied for processing polycrystalline diamond without binder (binderless PCD) in this current work. Our described innovative Ultra High Pressure Spark Plasma Sintering (UHP-SPS) equipment shows the combination of our high-pressure apparatus (Belt-type) with conventional pulse electric current generator (Fuji). Our UHP-SPS equipment allows the processing up to *6 GPa*, higher pressure than HP-SPS equipment, based on a conventional SPS equipment in which a non-graphite mold (metals, ceramics, composite and hybrid) with better mechanical properties (capable of *1 GPa*) than graphite. The equipment of UHP-SPS and HP-SPS elements (pistons + die) conductivity of the non-graphite mold define a Hot-Pressing process. This study presents the results showing the ability of sintering diamond powder without additives at *4–5 GPa* and *1300–1400 °C* for duration between *5* and *30 min*. Our described UHP-SPS innovative cell design allows the consolidation of diamond particles validated by the formation of grain boundaries on two different grain size powders, i.e., *0.75–1.25 μm* and *8–12 μm*. The phenomena explanation is proposed by comparison with the High Pressure High Temperature (HP-HT) (Belt, toroidal-Bridgman, multi-anvils (cubic)) process conventionally used for processing binderless polycrystalline diamond (binderless PCD). It is shown that using UHP-SPS, binderless diamond can be sintered at very unexpected P-T conditions, typically ~*10 GPa* and *500–1000 °C* lower in typical HP-HT setups. This makes UHP-SPS a promising tool for the sintering of other high-pressure materials at non-equilibrium conditions and a potential industrial transfer with low environmental fingerprints could be considered.

## 1. Introduction

Spark Plasma Sintering (SPS) is a well-known sintering technique born in Japan in the 60s that consists of using pulsed current injected into a powder to be sintered [1,2]. Since then, a lot of development through different patents [3] has been made and a different generation of SPS succeeded to the current fifth generation of SPS that allows industrialization of the process [3,4].

**Figure 1:**
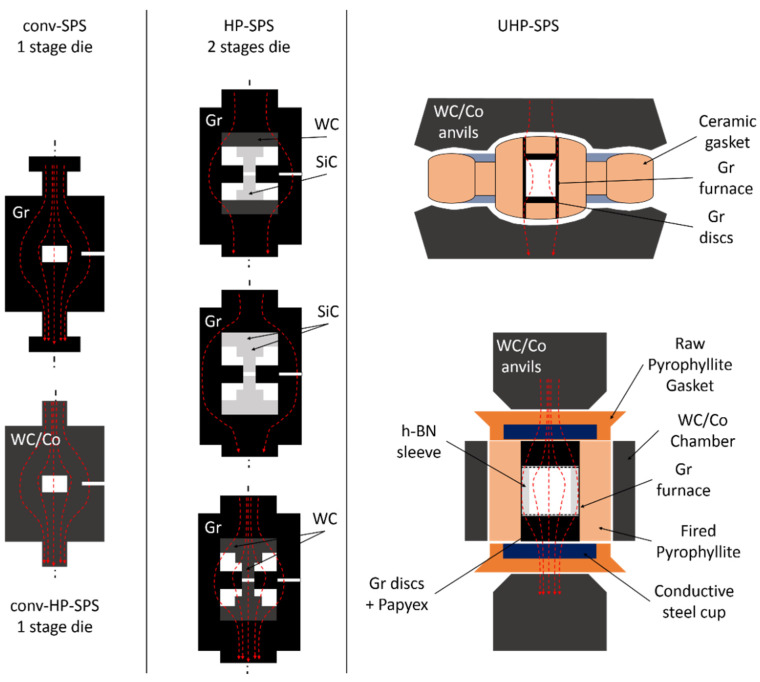
Drawing representing the different SPS assemblies and pathways of the injected current (red arrows). Conv-SPS assemblies (**left columns**) showing the mold contents, the sample and the furnace. These assemblies consist of mold mostly in graphite (Gr) to reach *P_max_* = *0.1 GPa* and *T_max_* ~ *2500 °C* and for HP-SPS in WC/Co to reach *P_max_* = *1 GPa* at low temperature (*100–200 °C*) (higher temperature for lower pressure is also possible). HP-SPS special assemblies (**middle columns**) in conventional equipment are more sophisticated and consist of double stage mold with an outer shell (acting as furnace) and inner die in graphite with hard materials discs/pistons (SiC and or binderless WC). In that case, *P_max_* = *1 GPa* and *T_max_* = *1100 °C* can be reached but the current pathway depends on the composition of pistons and discs. Therefore, the SPS process does not necessarily occur. In UHP-SPS setups (**right column**), assemblies are much more complicated to ensure good hydrostaticity. Typically, a ceramic cell is used to receive a thin wall graphite furnace in which powder is loaded. Powder is then packed between graphite punches inside the graphite tube furnace that allow current injection inside the sample (SPS principle). Hence, *P_max_* = *6–8 GPa* and *T_max_* = *2000 °C* can be achieved.

Conventional SPS (conv-SPS) is widely used in materials sciences [4] and is starting to be used by experimental petrologists [5]. Typically, suitable materials for SPS are metals, ceramics, composites and even organic materials. Basically, the principle of SPS is similar to that of hot pressing (HPing), the main difference being the heating procedure of the starting materials. Powder is loaded into a graphite die and enclosed by two graphite punches (Figure 1). Rarely, powder is packed in WC/Co die and pistons for higher pressure but lower temperature purposes (Figure 1). The assembly is then placed between two electrodes that are used to apply pressure (typically up to *100 MPa*) and to inject the current through the graphite (WC/Co) mold and the powder. It is of note that graphite or WC/Co ensures hydrostaticity and acts as furnace. Heating occurs therefore by the Joule effect and the injected current inside the powder has a significant effect that depends on the materials. Typically, for non-conductive materials, the electric charge accumulates at the surface particles, favoring surface diffusion, particles breakdown and formation of grain boundaries [6,7,8]. As a consequence, densification occurs at a lower temperature.

Pressure *(P)* is, like temperature *(T)*, a fundamental driving force that can favor a lot of different physical and chemical processes that can optimize properties of materials such as mechanical, optical and thermal.

**Figure 2:**
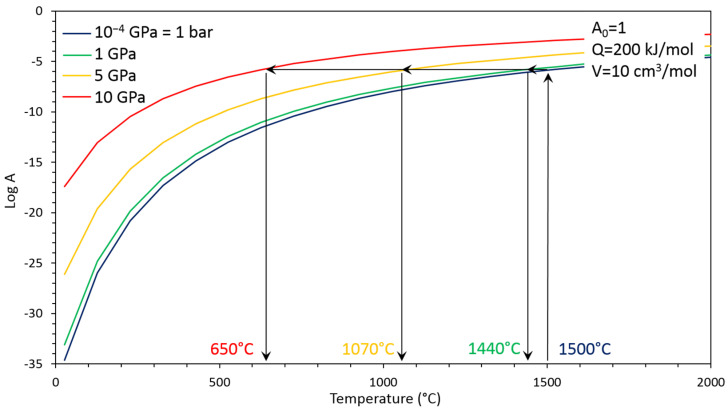
Graphic illustration of Equation (1) showing the efficiency of pressure (colored curves) to activate a physical or chemical process *A* as a function of temperature. Starting parameters *A*_0_, *Q* and *V* have been chosen arbitrary for illustration purposes. Sintering can be activated at several hundred degrees lower when applying high pressure of few GPa than at 1 bar (*10^−4^ GPa*).

Indeed, a lot of different physical and chemical processes obey to a temperature and/or pressure activation that can be described for example as an Arrhenius-type equation (Figure 2):

(1) $$A=A_{0}\times e^{\left(-\frac{Q+PV}{RT}\right)}$$

where *A* is the process, that can be for example, a diffusion coefficient, a grain growth rate, a grain boundary formation rate, a reaction kinetic, etc. In this Equation (1), *A*_0_ is pre-exponential term, *P* is the pressure (*in Pa*), *T* is the temperature (*in K*) and *R* the perfect gas constant (*R* = *8.314 J mol^−1^ K^−1^*). The most interesting terms here are the activation energy (*Q in J mol^−1^*) and activation volume (*V in m^3^ mol^−1^*). Thus, high pressure can start the activation of a given process *A* at several hundred degrees lower than at ambient pressure (Figure 2).

In particularly, pressure allows the synthesis of materials, even with different thermal stabilities precursors [9]:

− to orientate the chemical reaction in the direction of synthesis leading to the densest phase by the Le Chatelier principle (ex: synthesis of diamond to the detriment of graphite), e.g., [10,11];
− to initiate a new finer microstructure by driving the phase transformation in polymorphic materials (ex: Al_2_O_3_: γ → α), e.g., [12,13];
− to improve the chemical reactivity for refractory materials sintering (borides, nitrides, carbides) to better densification compared to lower pressure processes, e.g., [14,15];
− to allow the sintering beyond the thermal decomposition temperature by the condensation effect, i.e., pressure stabilizing structure (ex: MgB_2_), e.g., [16];
− to sinter the high-pressure stable phase in the high-pressure stability domain (ex: c-C, c-BN), e.g., [17];
− to adjust the porosity, close to 0% (ex: transparent ceramics) or high porosity (*p > 50%*) (ex: bone structure mimetic), e.g., [18];
− to increase the thermal stability of precursors by condensation effect by avoiding the departure of OH^−^, H_2_O, others volatile elements) [19];
− to decrease the sintering/consolidation/densification temperature by its driving force in order to avoid grain growth (which is always activated by high temperature), e.g., [20];
− to favor the structural phase existing only at lower temperature (ex for amorphous calcium phosphate), e.g., [21];
− to allow the consolidation of thermally unstable materials such as organic materials (ex: polymer) [22] and to allow the consolidation of composite constituted by materials of different thermal stability (ex: polymer composites) [23].

In this study, we therefore focus on the combination of high pressure (HP) with SPS, the technical developments and relevant results that have been made and obtained in the last decade. We will develop in more details the very new Ultra-High-Pressure (UHP)—SPS equipment and the promising results on the sintering of binderless polycrystalline diamond (binderless PCD) at very unexpected conditions highlighting the very high potential of this new technique compared to the classical PCD containing cobalt (Co-PCD) and their limited properties (Section 4).

## 2. Development of High Pressure SPS (HP-SPS) Using Conventional Equipment

**Figure 3:**
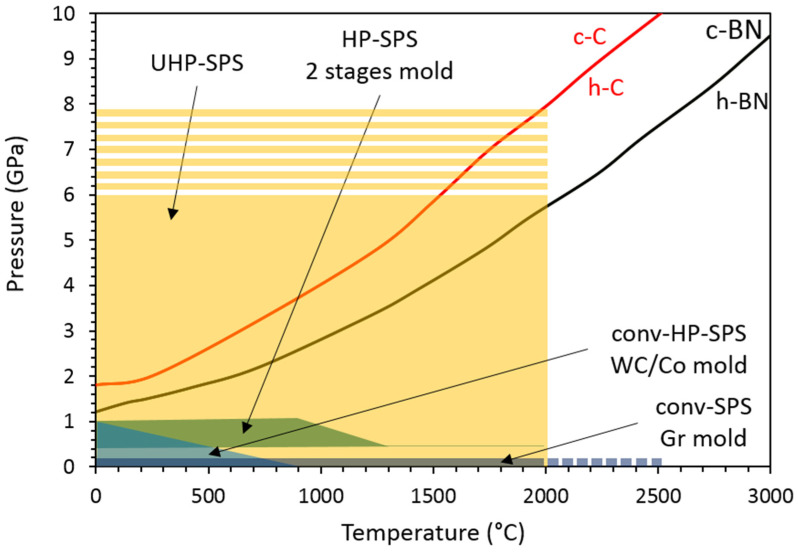
Pressure–temperature diagram showing filed of applications of the different SPS setups. Conv-SPS and HP-SPS are both used in conventional equipment and never exceed *1 GPa*. Maximum temperature depends a lot on materials constituting the mold, i.e., graphite, WC/Co, WC or SiC. In UHP-SPS equipment, a wide range of pressure–temperature conditions are covered offering the possibility to study sintering behavior of high-pressure phases such as c-C (red-curve) and c-BN (black curve) without any additions of binder.

Effect of pressure using conventional 1-stage assembly (Figure 1) was already observed despite the small range of pressure achievable with graphite mold (Figure 3) or higher pressure with much lower temperature using WC/Co mold (Figure 1 and Figure 3) [24,25]. Typically, pressure decreases sintering temperature and therefore limits grain growth.

In order to further inhibit grain growth during SPS process by reducing sintering temperature and therefore to keep a nano grains microstructure and enhance mechanical properties of materials due to Hall–Petch effect, some developments were made to perform high pressure experiments in conventional SPS equipment.

In 2006, the first double-stage SPS assembly was designed to perform experiments at pressure of up to *1 GPa* and temperature to *930 °C* [26] (Figure 3). Technical developments involved the use of an outer (1st stage) and inner (2nd stage) graphite stage (Figure 1). This outer graphite stage (heater shell) is used to ensure good heating rate. The other parts of the second stage consist of hard material pieces such as homemade binderless WC spacers and SiC pistons. By using this double stage assembly, samples are very small, typically 5 mm in diameter and *1* to *3 mm* thick. With this setup, it was shown that *95%* relative density of fully stabilized zirconia is achieved at *900 °C* at 1 GPa whereas *1400 °C* is needed at *40 MPa*. Therefore, this setup showed the efficiency of HP-SPS by keeping a microstructure with *10 nm* grains at *1 GPa*, *900 °C*, whereas they grow to *200 nm* at *40 MPa* and *1400 °C*. Similar setup was also used by replacing binderless WC protective discs by SiC ones [27] or simply by removing binderless WC discs [28] (Figure 1). In both cases, *P* ~ *0.5 GPa* and temperature up to *1200 °C* were reached (Figure 3), improving mechanical properties and transparency of sintered nano zirconia [27] and nano α-Al_2_O_3_ (corundum) [28]. Finally, other tests were performed by replacing inner graphite die by SiC ones [29,30,31]. With these modifications, sample diameter can reach 10 mm and still few mm thick. Again, maximum conditions are *P* ~ *1 GPa* and *1000 °C* whereas for lower pressure (*0.4 GPa*), temperature can be increased to 1300 °C (Figure 3), conditions that are sufficient to sinter nano grain transparent MgAl_2_O_4_ spinel [29,30] and α-Al_2_O_3_ [31].

Some other modifications of this pioneering double-stage assembly for HP-SPS in conventional equipment were proposed. In more details, SiC pistons were replaced by binderless WC ones [32,33,34] (Figure 1). Pressure and temperature conditions were not necessarily enhanced by these modifications and typically, maximum conditions were *P* ~ *0.5* *GPa* and *950–1000 °C* even theoretically pressure of 1 GPa and temperature up to *1200 °C* could be reached (Figure 3). In any of these studies, nano-grained transparent materials are obtained.

However, by comparing these two types of pistons, the question about the heating and current pathways aroused. It is of note that no information is given about the structure of the SiC used (α vs. β) which asks the questions whether there are conductive or insulators and that therefore if current passes through the powders (SPS principle)

Indeed, electrical resistivity of SiC is about 8–9 order of magnitude higher than that of binderless WC, *10^2^–10^3^ Ω m* and *2.0 × 10^7^ Ω m*, respectively [32]. This would imply that using SiC pistons and/or discs, samples would be electrically isolated, that heating would occur by resistive heating of the outer and inner graphite dies and that the spark effect of the SPS process would not occur between grains. Sintering would therefore occur by hot pressing (Figure 3). However, the use of binderless WC pistons and discs allows the current to be injected inside the sample powder and the SPS process is effective. Moreover, heating is not only ensured by the graphite dies but also by the pistons. Additionally, to reach higher temperature at relatively high pressure, low-cost carbon fiber composite (CFC) pistons were also developed and allow to reach *2000 °C* at *0.4 GPa* [34] (Figure 3).

To summarize, HP-SPS double stage assemblies using conventional equipment were essentially developed in order to decrease sintering temperature and therefore keep a nano-grain microstructure that enhance strengthening of materials due to the presence of high density of grain boundaries, so called Hall–Petch effect. However, by using these double stage assemblies with always an outer graphite die, pressure and temperature are limited to *1 GPa* and *1000 °C*, respectively (Figure 3). Moreover, materials used as pistons asked the question of real SPS process inside the samples (Figure 1). There is therefore a real lock to overpass with the development new material molds with significant electrical conductivity and also high mechanical properties that would allow to work at both high-pressure high temperature and also under air conditions instead of vacuum. Finally, in order to consider sintering by SPS of very hard materials such as diamond and c-BN, higher pressure and temperature are mandatory and new equipment have to be developed (Section 3).

## 3. Recent Developments: UHP-SPS

Based on the respective advantages of high pressure and spark plasma sintering, i.e., lowering temperature and faster conditions of sintering as well as the stabilization of high-pressure phases, two experimental setups were recently designed to couple Ultra-High-Pressure apparatus (UHP) and Spark plasma sintering (SPS), a process so-called UHP-SPS that can reach up to *6* to *8 GPa* and *2000 °C* (Figure 3).

Two different groups were coupled with ultra-high pressure (UHP) with SPS in the past 5 years. In both groups, a large volume press was used with their typical assembly. The main difference with conventional 1- or 2-stage SPS is that in large volume press assembly, furnaces use thin wall graphite, metals or LaCO_3,_ whereas the whole mold is the furnace in conventional SPS equipment (1- and 2-stage) (Figure 1). Therefore, less energy is needed to heat these thin wall furnaces compared to large molds. It is also of note that no vacuum is needed by using these UHP-SPS setups contrary to conventional SPS and HP-SPS in conventional equipment. Indeed, contrary to conventional SPS or even HP-SPS using graphite dies, graphite furnace and spacers in UHP-SPS are never in contact with air but enclosed in ceramic pressure transmitting medium and during the process no oxygen is available

The first group, from the University of Krakow, Poland, coupled a Bridgman type toroid large volume press and a homemade *50 Hz* alternating or *1 kHz* pulsed current injected inside the sample thanks to an adapted designed assembly (Figure 1) [35,36,37]. Typically, assembly consists of a pressure transmitting medium (ceramic) in which a graphite furnace is inserted in which sample (*15 mm* diameter and *5 mm* thickness) is loaded at the center. Sleeves and spacers are typically used to improve good hydrostaticity and the specificity here is that graphite spacers are inserted at the top and bottom of the sample and in contact with the furnace to allow the current to be injected in the sample and therefore create and use the SPS process (Figure 1). Using this setup, very refractory phases were sintered and the combine effect of pressure and SPS allows to decrease sintering temperature and time, and therefore maintain a nano-grain structure with enhanced mechanical properties [35,36]. Another study compared classic HP-HT (sample electrically isolated) and UHP-SPS of diamond-TiB_2_ composite under similar conditions of pressure and temperature [37]. Due to the advantages of SPS, diamond remains stable whereas it destabilizes to graphite using classic HP-HT [37], making UHP-SPS a promising tool to sinter high pressure phases even at metastable/unequilibrium conditions.

The second group, from the University of Bordeaux, France, associated a Belt-type large volume press and a conventional electric pulsed current source from Fuji Electronic Industrial (model SCM-3000) that can reach *3000 A* for *10 V* with a pulse duration of *3 ms*. Details on the press design and current source as well as calibration are available in [38]. Assembly is an external pressure transmitting medium in fired pyrophillite in which a graphite furnace is inserted (Figure 1). Sample powder (maximum *17 mm* diameter and *10 mm* thickness) is loaded directly in contact with the furnace or surrounded by a sleeve, mostly in h-BN (Figure 1). Powder is directly in contact with graphite punches to allow the injection of current (Figure 1). By using this setup, is has been shown that the direct conversion sintering of γ-Al_2_O_3_ to α-Al_2_O_3_ occurred at much lower temperature than is classic HP-HT setup, for example at *500 °C* and *800 °C* at *1.5 GPa* [12,38,39]. This setup was also used for the joining/consolidation of drilling bits at high pressure temperature by joining WC/Co substrate tables by polycrystalline diamond compact (PDC: Diamond/Co and pure diamonds) [38]. In both cases, sintering occurred at lower temperature than in classic HP-HT setups. Another example showed the sintering of MgB_2_ [16]. Here, the increase in pressure (in the range *2* to *5 GPa*) stabilized the phase above its decomposition temperature and high temperature promoted the sintering up to relative density of 100% with a fine-grain microstructure [16].

These two setups were compared with conventional SPS for the sintering of β-SiC [40]. No obvious difference was observed between the two UHP-SPS setups. However, it is clear that the high pressure promotes the densification at lower temperature, therefore limiting diffusion and associated grain growth that could occur in conventional SPS due to the very high temperature of sintering (*T* ~ *1800 °C*) [40].

A final attempt was to use cubic-type multi-anvil press with an adapted assembly to allow an AC current to be injected inside the sample [41]. In this way, pure Mo sintering was achieved up to *98.5%* at 9 GPa, *1300 °C* and for *1 min* [41]. Pressure is the main parameter to obtain highly dense materials and injection of current inside the samples has a benefit effect on hardness and bending strength, as well as on limited grain growth due to the fast process [41].

## 4. Application to Binderless Diamond Sintering

### 4.1. Brief History of Binderless Diamond Sintering

Since the first synthesis of diamonds in the mid-50s, early 60s [10,11], a lot of setups were developed to sinter diamonds and fabricate bulk objects. In the 70s, polycrystalline diamond compacts (PDC) were processed with the help of a metallic binder [42]. Briefly, the principle is to synthesis diamond particles from graphite that is mixed with a metallic catalyzer (mostly Ni). At *4–5 GPa*, *1300–1400 °C*, graphite transforms to diamonds grains [43]. After recovery and different steps of leaching, these diamond grains are sintered at HP-HT using a Cobalt binder to make these PCD that are typically mounted on WC/Co supports to fabricate PDC [42]. Due to their good mechanical properties, these objects are used for cutting and drilling tools. However, above *400 °C*, these objects deteriorate quite easily due to heating by friction [44].

In order to overcome this issue, to reach higher mechanical properties with higher life duration tools and also to use other diamond aspects such as its optical or thermal properties, binderless diamond sintering has been developed almost 20 years ago from the pioneering work of Voronov (2000) and Irifune et al. (2003) [45,46].

A wide literature exists on binderless diamonds sintering and a recent review has also been published [47]. Therefore, in the following, a brief summary is given concerning the technical development, the physical process to sinter these objects and their associated physical properties.

Typically, there are two methods to sinter binderless diamonds using High Pressure—High Temperature (HP-HT) techniques. In both methods, quasi-hydrostatic pressure is generated using a specific hydraulic press called a multi anvil apparatus (MAA) that can generate very high pressure (several tens of GPa typically) on large samples (few mm^3^) due to their multi-stage assemblies. Temperature is typically generated using resistive heating furnaces in graphite or metals thanks to a high-power power supply. Finally, samples are thermally isolated from the furnace using specific materials.

The first method was jointly developed in Japan by Geodynamics Research Center (GRC) and Sumitomo Electric Industry (SEI), represented by Pr. T. Irifune and Dr. H. Sumiya, respectively, and consists of Direct Conversion Sintering (DCS) of graphite [46,48,49,50,51,52,53,54,55] or other pure carbon sources [49,51,56] to diamond objects. In order to obtain an object of ~*1 cm^3^*, dedicated Kawaï multi-anvil press was developed [57]. Typically, DCS occurs for diamond at *P* > *15 GPa* and *2000 °C* < *T* < *2500 °C* and final products are yellowish (due to the presence of nitrogen) transparent Nano-Polycrystalline Diamond (NPD) compact. According to the P-T conditions and carbon source, Lonsdaleite, another high-pressure phase of carbon, and lamellae structures are more or less observed, that can modify physical properties of the NPD [49,51]. Typically, NPD have exceptional mechanical properties (Young’s modulus, hardness, wear rate…) that highly exceed those of PDC and even those of natural diamonds single crystal [50]. Optical and thermal properties are also exceptional and similar to those of natural single crystal [58]. Therefore, NPD are the perfect tools that can replace PDC and natural single crystal for cutting/drilling tools, optical windows and thermal sink. However, the very high P-T conditions and very specific equipment make the industrial transfer impossible and up to now, applications are essentially dedicated to scientific areas [59].

The second method tries to decrease sintering conditions of binderless diamonds using directly diamonds powders. This protocol was first trialed by Hall, 1970 [17] who argued that diamond powders can be sintered at high pressure-high temperature. The degree of sintering depends on P, T, t pathways as well as on starting grain size [17], an idea that was developed later with the addition of the nature of precursors (natural, HPHT and detonation diamonds) [45,60,61]. However, sintered objects were never fully dense, and some back transformation occurred due to the limited P-T conditions reached in their respective apparatus (piston-cylinder and belt-type press). This method was further developed by the University of Sichuan, China [62,63,64,65,66]. An optimized cubic hinge-type apparatus was used to sinter objects up to *0.5 cm^3^* at pressure up to 16 GPa and temperature to *2500 °C* [62]. Fully dense diamond objects, called CF-PDC or MPD for Catalyst-free Polycrystalline Diamonds compact or Microcrystalline Polycrystalline Diamond, can be obtained at pressure and temperature down to *14 GPa* and *1500 °C*, respectively [63,64,65,66]. However, samples that present micro-cracks are black translucent, and TEM observations revealed the presence of a very small amount of graphite at triple junctions [63,64,65]. Despite these features, hardness of CF-PDC or MPD are similar to those of NPD and present a high thermal stability, but the optical properties are not very useful [63,64,65].

### 4.2. UHP-SPS Setup with Belt-Type HP Apparatus for the Sintering of Diamond Powders

One of the main goals to couple UHP and SPS is to sinter high pressure phases at unexpected conditions of pressure and temperature (Figure 3) without the use of any binder to ensure exceptional physical properties (mechanical, optical, electrical and thermal). In this aim, one can site the sintering of very hard materials such as c-BN and diamonds (c-C) that are stable at pressure well above those achievable by conventional SPS and 2-stage HP-SPS, i.e., limited to maximum pressure of 1 GPa. As described above, these two materials are sintered at minimum pressure of *8–9 GPa* for c-BN and *12 GPa* for diamonds using multi-anvil devices and by resistive heating [52,67,68].

**Figure 4:**
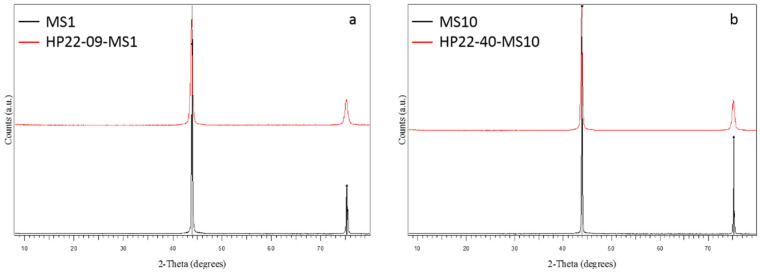
XRD patterns of starting materials (black) and run products after experiments (red): (**a**) For the experiments conducted with *0.75–1.25 μm* grain size powder and (**b**) with *8–12 μm* grain size powder. The starting powders are very pure, and grains are well crystalized (sharp peaks). After experiment, diamond is fully conserved with no evidence of graphite back transformation. It is of note that diffraction peaks in experimental products are wider than in the starting powder.

**Figure 5:**
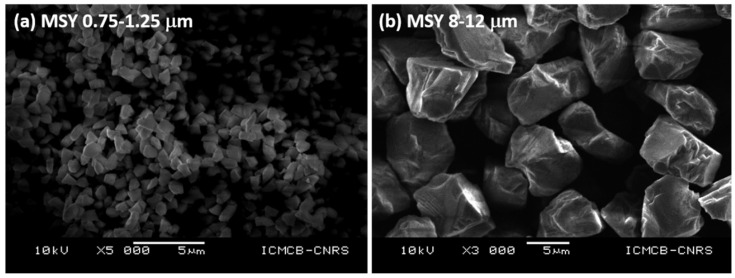
SEM images (SEI mode, *10 kV*, WD = *10 mm*) of the starting powders: (**a**) *0.75–1.25 μm* grain size and (**b**) *8–12 μm* grain size.

Here, sintering of binderless diamonds were performed using two different grain sizes of commercial diamonds from the company Pureon. These powders are synthetic diamonds produced by HP-HT techniques using metallic catalyst and purified by chemical treatments (MSY grade, purity > 99%) and grain sizes were precisely controlled and are *0.75–1.25 μm* and *8–12 μm* with a narrow gain size distribution. XRD show only sharp diamond peaks, typical of very well crystallized powders (Figure 4). SEM images show automorph grains, and average grain sizes correspond to those provided by the supplier. (Figure 5).

**Figure 6:**
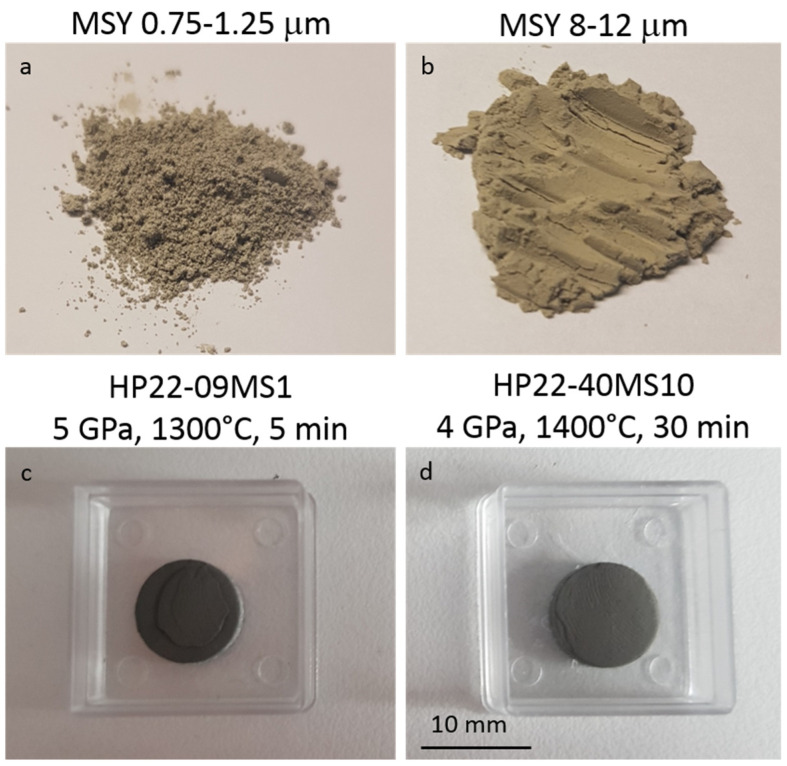
Macroscopic view of starting materials (**a**,**b**), with grain size *0.75–1.25 μm* (**a**) and *8–12 μm* (**b**), respectively. Starting powders are yellowish. Bottom row shows recovered samples after experiments at HP-HT using our UHP-SPS setup and powders described above (**c**,**d**). Recovered samples are solid discs of *11 mm* diameter and *2–3 mm* thickness.

For each run, about 0.5 g of diamond powder was packed in the UHP-SPS assembly. It is of note that for contamination/pollution purpose, powders were not packed directly in contact with the graphite furnace and papyex sheets. For this purpose, a sleeve of h-BN and two Mo discs are placed at the top and bottom of the powder (Figure 1). This reduces the sample size to *11 mm* in diameter and *2–3 mm* in thickness (Figure 1 and Figure 6c,d).

**Figure 7:**
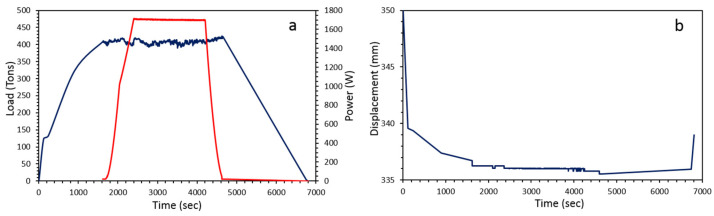
(**a**) Load (tons, in blue) and power (W, in red) parameters as programmed and recorded during experiments and associated displacement in mm (**b**). During dwell, both load and power, i.e., pressure and temperature, are very stable. Most of the displacement occurs during cold compression (few mm) whereas it is only of few tens to hundreds of microns during heating and dwell.

For each run, pressure is first increased to the target in *30* to *45 min* using calibration made on the variation of resistance of different pressure metal calibrant that experienced phase transition at a given pressure [38]. Then, temperature is increased to the target based on the previous calibration made on the melting point of temperature metals calibrant at *4 GPa* [38]. Heating rate is typically *100 °C/min*, using a SPS pulsed current of *3.3 ms* and a *ON:OFF* sequence of *12:2*. Dwell times were varied from *1* to *30 min* and decompression was also varied from *30 min to several hours* (overnight) (Figure 7a). During the whole process, anvil displacement is recorded first for follow powder densification and also to ensure that there is no contact between anvils and WC/Co die (chamber, cylinder, ring) (Figure 7b).

**Figure 8:**
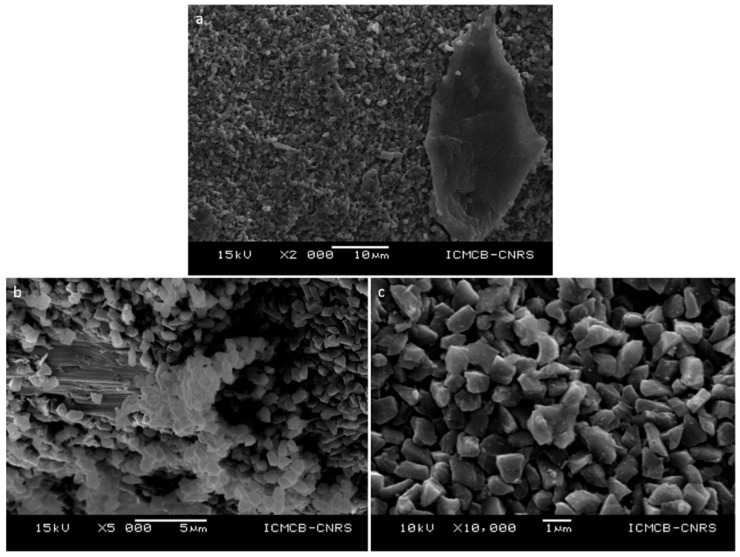
Microstructure of HP22-09-MS1 recovered sample observed in SEM (SEI, *15 kV*, WD = *10 mm*) at different magnification ((**a**) ×2000, (**b**) ×5000 and (**c**) ×10,000). First, there is no evidence for grain fracturation, nor the presence of other phases. There are two types of regions inside the samples (**a**,**b**), typically more or less sintered suggesting that densification is heterogeneous and not total (**a**–**c**). In more densified areas, a lot of grain boundaries are observed (**b**) whereas grains are only packed in less densified areas with only few grains that can be sintered (center of (**c**) for example).

**Figure 9:**
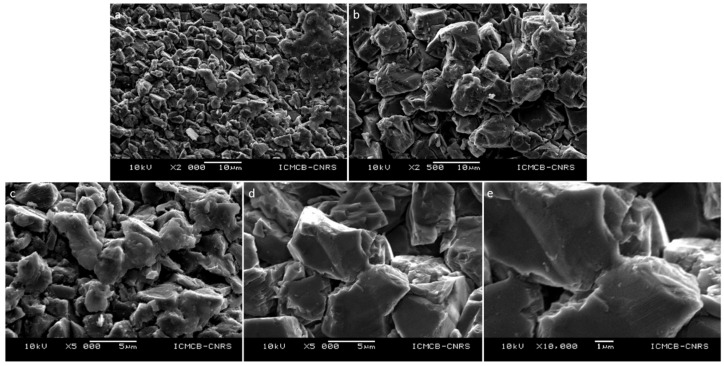
Microstructure of HP22-40-MS10 recovered sample observed in SEM (SEI, *10 kV*, WD = *10 mm*) at different magnification ((**a**) ×2000, (**b**) ×2500, (**c**,**d**) ×5000 and (**e**) ×10,000). There is no evidence for grain fracturation, nor the presence of other phases. Due to larger grain size, there are less areas sintered (**a**,**b**). However, in these areas, it is evident that grains boundaries are formed or in the process of formation (**c**–**e**).

Two experiments are presented in the following. Run HP22-09-MS1 was performed using *0.75–1.25 μm* powder loaded at *5 GPa* and heated to *1300 °C* for *5 min* and run HP22-40-MS10 was carried out *8–12 μm* powder at *4 GPa* and *1400 °C* for *30 min* (Table 1, Figure 4, Figure 6, Figure 8 and Figure 9).

After experiment bulk samples were recovered as solid discs of ~*11 mm* diameter and *2–3 mm* thick (no mass loss and no color change was observed compared to the starting powders (Figure 6)). Although experiments were performed at the limit or out of the diamond stability field, for both recovered samples, XRD patterns show only diamond peaks with no evidence of destabilization into graphite or more amorphous sp2 forms (Figure 4). The only difference with XRD of the starting powders is that diffraction peaks are wider after experiments (Figure 4). It is unlikely to be due to a grain size reduction during experiments as shown on SEM images where no or little grain fracturation and grounding are observed (Figure 8 and Figure 9). Therefore, it is most likely to be due to residual stress inside grains after experiments.

Sample density was measured using the Archimedes method. It is found that HP22-09-MS1 has a density slightly higher than HP22-40-MS10 at *3.05 g cm^−3^* and *2.80 g cm^−3^*, respectively, corresponding to a relative density of *87* and *79%* or a porosity of *13* and *21%*. This suggest that is easier to sinter small grain size powder of around 1 micron than a bigger grain size around 10 microns. Despite this relative low density, samples are very mechanically resistant. It is of note that density can be improved by mixing different sizes of diamond powder and plays on the P, T, t pathway.

These compacts, which were not fully dense, were selected to show better images of the neck formation between the grains. SEM observations using a JEOL-6360A (SEI mode, *10–15 kV*, WD = *10 mm*) at low magnification (×2000) show different areas more or less densified inside both samples (Figure 8a and Figure 9a,b). Indeed, it is clearly visible that some areas are “only” compacted powders whereas others show sintered structures. This suggests that there is a kind of heterogeneity inside the samples. This could be due to local thermal/pressure gradient during the sintering process and/or that conditions of duration are not optimized to fully densified samples. It is also observed that there are more dense areas in samples sintering with *0.75–1.25 μm* grains (Figure 8a) than in the *8–12 μm* grains (Figure 9a,b), explaining the difference in the measured density (Table 1). At higher magnification (×5000 and ×10,000), grains are more visible, no grain fracturation is observed in both samples and 1-micron grains have still sharp edges compared to starting powder, whereas those of 10-microns grains are smoother than in the starting powder. Moreover, two different types of microstructures are clearly present in both samples (Figure 8b,c and Figure 9c–e). On one hand, samples are composed of compacted grains, i.e., as a green body. On the other hand, the denser areas in both samples show the formation of grain boundaries between grains (Figure 8b,c and Figure 9c–e) at very unexpected pressure-temperature conditions. These dense areas are more present and in samples containing 1-micron grains (Figure 8b,c, HP22-09MS1) than in 10-microns grain sample (Figure 9c–e, HP22-40-MS10). Indeed, in the former case (1-micron grains) hundreds to thousands of grains could be sintered together forming these large dense areas (Figure 8b) whereas in the latter case (10-micron grains), only few grains are sintered together (Figure 9c–e). It of note that small powder grain sizes were sintered at higher pressure (5 GPa) and lower temperature (*1300 °C*) and for shorter duration (*5 min*) than the bigger powder grain size (*4 GPa*, *1400 °C*, *30 min*). This indicates that pressure is of main importance in the sintering process and that smaller grain size diamond powder are easier to sinter compared to bigger ones. It could therefore be very interesting to sinter nanodiamonds at similar conditions to improve densification. However, as sintering conditions are close or even out of the stability field of diamonds, there is subtle balance to consider between powder grain size, its reactivity and P-T conditions.

These results show that, by combining high pressure apparatus and SPS, the sintering of high-pressure phases such as binderless diamonds could be achieved at much lower conditions than those previously conducted by conventional HP-HT apparatus. This opens a new field in material sciences for the very hard materials sintering and the stabilization of high-pressure phases.

## 5. Conclusions

Conventional SPS has been used for decades for the sintering of very different materials such as metals, ceramics or composites. The main advantages of this technique are twofold. The high temperature is reached quickly and the current injection inside the powder allows a fast densification of samples. Thus, grain growth is inhibited, and grain size remains relatively small, improving materials properties.

With the aim to further enhance these properties, HP-SPS setup has been developed to be adapted in conventional equipment. Two-stage assemblies with hard materials pistons (in SiC or pure WC) increase pressure conditions to *1 GPa* but temperature cannot exceed *1000 °C* and sample sizes are also limited to few mm in diameter and thickness. However, the high pressure has the effect to reduce the sintering temperature and therefore samples with nano-grains (few nm to few tens of nm) microstructure were obtained. Recovered samples have typically better mechanical and optical properties than those sintered by conventional SPS.

The recent developments of UHP-SPS equipment with dedicated large volume press (Bridgman or Belt-type apparatus) allows reaching *6* to *8 GPa* and up to *2000 °C*. Such high pressure still decreases sintering temperatures and coupling with SPS yields similar results compared to classic HP-HT. Moreover, such high pressures allow to stabilize phases with low temperature destabilization and also promote the densification of high pressure phases such c-C without any binder at very unexpected conditions. Indeed, we have shown that microcrystalline diamond powders (*0.75–1.25* and *8–12 μm*) could be sintered at *4–5 GPa* and temperature between *1300* and *1400 °C*. Although samples are not fully dense, observation of grain boundary formation highlight the sintering process at P-T conditions out of diamond equilibrium. Some improvements can be made to obtain fully dense materials such as dwell duration, heating, cooling and decompression rate, mixing of different grain size powders and probably the on:off sequence. UHP-SPS is thus a promising tool that could be applied to a lot of different hard materials such as c-BN or other borides, nitrides, carbides and even hard ceramics and composites.

Finally, environmental fingerprints are reduced by using these high pressure SPS sintering because they consume less electrical energy and materials obtained have better properties providing them a high life duration.

## Figures and Tables

**Table 1:** Experimental conditions and main results.

Run #	Grain Size (μm)	Quantity (g)	P (GPa)	T (°C)	Dwell Time (min)	^1^ Density (g cm^−3^)	XRD	Grain Boundary Formation
HP22-09-MS1	0.75–1.25	0.5	5	1300	5	3.05	c-C	+++
HP22-40-MS10	8–12	0.5	4	1400	30	2.80	c-C	++

^1^ Density measured by Archimedes’ method.

## Data Availability

Not applicable.
